# Gestational age at birth, birth weight, and gestational age when intrauterine brain sparing occurs determines the neonatal outcome in growth-restricted infants born before 32 weeks of gestation: a retrospective cohort analysis

**DOI:** 10.3389/fped.2024.1377982

**Published:** 2024-07-04

**Authors:** Franziska Köber, Yvonne Heimann, Thomas Lehmann, Ekkehard Schleußner, Hans Proquitté, Tanja Groten

**Affiliations:** ^1^ Section of Neonatology, Department of Pediatrics, University Hospital Jena, Jena, Germany; ^2^ Department of Obstetrics, University Hospital Jena, Jena, Germany; ^3^ Institute of Medical Statistics, Information Sciences and Documentation, University Hospital Jena, Jena, Germany

**Keywords:** fetal growth restriction, preterm birth, very low birth weight, very preterm birth, brain sparing, pentaerythrityl tetranitrate (PETN)

## Abstract

**Background:**

Preterm birth and fetal growth restriction are the main determinants of perinatal mortality. In the absence of therapeutic interventions, management is restricted to the observation of fetal growth and fetoplacental perfusion to determine the timing of delivery. Fetal circulatory redistribution, known as “brain sparing,” represents a sign of fetal hypoxia and has been implemented in algorithms for when to deliver. In the absence of any other option, the nitric oxide donor pentaerythrityl tetranitrate (PETN), which has been shown to improve fetoplacental flow and reduce preterm birth in high-risk patients, is offered to patients as a personal therapy attempt. The aim of this study was to evaluate determinants related to pregnancy, including PETN intake during pregnancy, on immediate neonatal outcomes in a cohort of growth-restricted infants born before 32 completed weeks of gestation.

**Methods:**

We performed a retrospective cohort study of 98 infants born with a birth weight below the 10th percentile before 32 completed weeks of gestation at our tertiary care center between 2010 and 2019. PETN was offered to all mothers with a history of severe adverse pregnancy outcomes who were at high risk of developing fetal growth restriction as an individual therapy attempt.

**Results:**

The mean gestational age at birth was 188.5 days, and the mean birth weight was 549 g, corresponding to a median percentile of three. In 73 (79.3%) cases, brain sparing occurred during pregnancy. A total of 22 (22.4%) neonates were stillborn, 20 died postnatally, and 37.3% developed a severe complication. Multivariable analysis revealed birth weight percentile, gestational age at birth, and gestational age when brain sparing first occurred to be robust predictors of mortality or severe neonatal morbidity. In 39 neonates of mothers taking PETN, this impact of brain sparing was not observed.

**Conclusion:**

Our study is the first to demonstrate a significant association between the early occurrence of brain-sparing and severe neonatal outcomes in a cohort of very early preterm, growth-restricted newborns. The data suggest that PETN intake may ameliorate the effect of brain sparing in the affected neonates.

## 1 Introduction

According to the World Health Organization (WHO), the global preterm birth rate remains at approximately 11%, and associated complications are responsible for an estimated 35% of the world's 3.1 million annual neonatal deaths. The WHO defines preterm birth as any birth before 37 completed weeks of gestation (1). Preterm birth can further be classified as extremely preterm before 28 completed weeks and very preterm before 32 completed weeks, while preterm birth after 32 weeks is denoted as moderate or late preterm. These subdivisions are important since decreasing gestational age is associated with increasing mortality and short-term and lifelong morbidity (2).

Immediate complications of prematurity include increased risks of neonatal respiratory conditions, such as respiratory distress syndrome, bronchopulmonary dysplasia (BPD) and pulmonary hypertension (PHT), necrotizing enterocolitis, sepsis, cerebral impairments, such as periventricular leukomalacia, intraventricular hemorrhage (IVH), cerebral palsy, and hypoxic-ischemic encephalopathy along with visual and hearing disabilities. Consequently, preterm birth has been linked to poor neurodevelopmental outcomes in childhood (3).

In addition to gestational age, birth weight, according to gestational age, is an important predictor of neonatal outcome. Infants who do not reach their genetically determined growth potential due to placental insufficiency leading to fetal growth restriction (FGR) experience significantly more complications compared to infants born at the same gestational age with an appropriate gestational weight (4). FGR is one of the most common pregnancy complications, affecting up to 10% of all pregnancies. The definition of FGR still varies between guidelines and countries. According to an international consensus published in 2016, FGR is defined as cases in which estimated fetal weight (EFW) and/or the abdominal circumference (AU) drop below the 3rd percentile or below the 10th percentile if signs of impaired fetal or placental perfusion are present. Impaired perfusion is established when the pulsatility index in the umbilical artery (UA-PI) and/or the mean PI of the two uterine arteries exceed the 95th percentile (5). FGR not only constitutes the main risk factor for stillbirth, but these infants have a fourfold risk of postnatal death and show worse neurodevelopmental outcomes with higher rates of conditions associated with prematurity, particularly respiratory distress syndrome and necrotizing enterocolitis (6). The detection of fetal brain sparing, defined as a relative increase in UA-PI and a corresponding decrease in PI in the arteria cerebri media (ACM-PI), represented as the cerebroplacental ratio (CPR), has been previously described as a predictor of perinatal outcome in late preterm fetal growth restriction (7, 8).

The nitric oxide (NO) donor pentaerythrityl tetranitrate (PETN) reduces the impedance in the uteroplacental vessels and has been shown to possess a protective effect on the maternal endothelium (9). In a randomized controlled pilot study, our group verified the beneficial effect of PETN on pregnancies, recognized by impaired uteroplacental perfusion at mid-gestation, being at risk for the development of FGR (10). In this study, the gestational age at which CPR dropped below the threshold of 1 was postponed in the PETN group (11). After the publication of our data, we offered PETN as an off-label individual therapy attempt to pregnant women with a particularly high risk of poor pregnancy outcomes due to FGR.

The aim of this study was to investigate prenatal features influencing the immediate perinatal and postnatal outcomes of infants born early, preterm, and growth-restricted. The factors analyzed included maternal demographic data, Doppler data, gestational age at birth, and birth weight, as well as the intake of PETN.

## 2 Patients and methods

**Figure 1:**
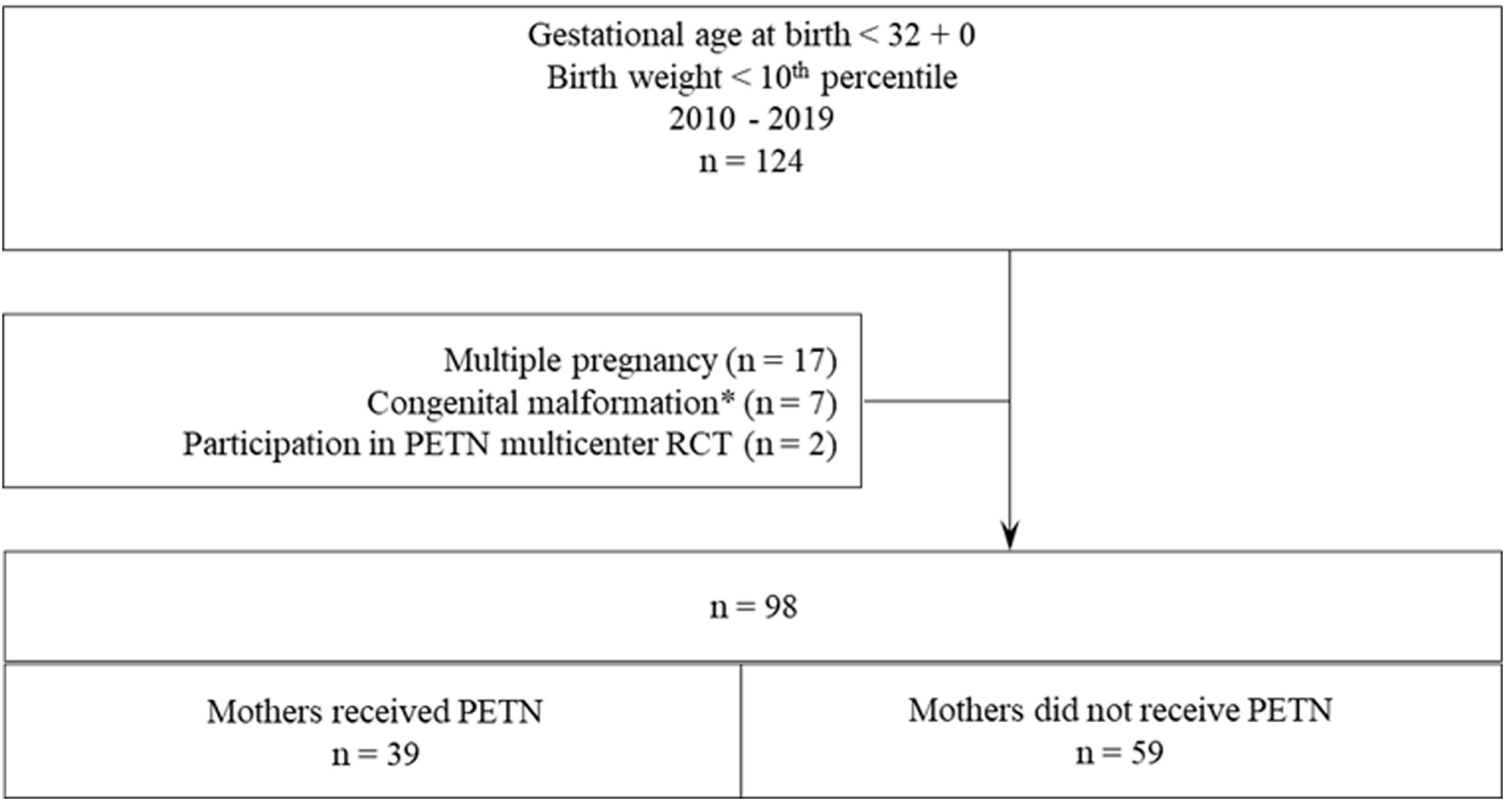
Cohort composition.

We conducted a retrospective single-center analysis of all infants born before 32 completed weeks of gestation with a birth weight below the 10th percentile (12) at our tertiary care hospital between 1 January 2010 and 31 December 2019. We observed a total of 124 newborns. After excluding non-singleton pregnancies (*n* = 17), cases with severe congenital malformations (*n* = 7), and 2 women who participated in the ongoing randomized blinded PETN multicenter trial, 98 cases were included in our analysis. High-risk pregnancies and neonates were cared for according to German guidelines. During the study period, mothers affected by FGR were offered PETN treatment as an individual therapy attempt. A total of 39 mothers requested PETN and took 50 mg twice a day (Figure 1). Ethical approval was obtained from the Ethical Committee of our institution (2019-1565-Daten).

Outcome data were retrieved from hospital records. Fetal biometry and Doppler measurements were performed using standardized anatomic views according to international society of ultrasound in obstetrics and gynecology (ISUOG) guidelines (13). Intrauterine percentiles were calculated based on gestational age and estimated weight using the HADLOCK 1991 formula (14). Reported Doppler parameters include uterine blood flow, the CPR calculated as the ratio of the PI of the arteria cerebri media and UA-PI (15), the absence of or reverse end-diastolic flow in the umbilical arteries (AEDF and REDF), and a negative A-wave in the ductus venosus. CPR was interpreted to be pathologic at a cutoff of 1 or lower, indicating cerebral redistribution of the fetus (16).

Neonatal outcome parameters reported are gestational age at birth; birth weight and birth weight percentile; pre- and postnatal death; any death (including pre- and postnatal death); IVH (any); severe IVH (grade III or III+); PHT (any); PHT requiring NO therapy; gastrointestinal complications requiring surgery; BPD; retinopathy of prematurity (ROP); and for survivors, the length of stay in the neonatal intensive care unit (NICU).

As the main outcome measures to describe neonatal mortality and morbidity, we chose three composite parameters. “Mortality or severe neonatal morbidity” describes any death or severe neonatal morbidity, “postnatal mortality or severe neonatal morbidity” describes a postnatal death before discharge and/or the occurrence of severe neonatal morbidity, and “severe neonatal morbidity” is reported for those who were discharged alive with severe neonatal morbidity. Severe neonatal morbidity was defined as the presence of severe IVH and/or gastrointestinal complications requiring surgery and/or PHT requiring NO therapy.

Statistical analysis was performed with SPSS 26.0. Statistical significance was defined as *p* ≤ 0.05. Since all analyses were exploratory, no correction for multiple testing was applied. Patient characteristics were reported as medians with 25th/75th percentiles for steady variables and as numbers of cases and relative frequencies for categorical variables. A chi-square test or Fisher's exact test was applied to compare categorical variables regarding the intake of PETN. A Mann–Whitney *U* test was performed to compare continuous variables between these two groups. Linear regression was applied for continuous outcomes, and logistic regression analysis was applied for binary outcomes to assess the influence of different independent variables.

## 3 Results

Group characteristics and neonatal outcome data are presented in Table 1. Of the neonates, 22 (22.4%) were stillborn, and 20 of the 76 live-born children died in the NICU before discharge, resulting in an overall death rate of 42 (42.9%). The median gestational age at delivery was 188.5 days (range: 22 + 0 to 31 + 6 weeks), and the median birth weight was 549 g (range: 195–1,290 g). The combined outcome of “mortality or severe neonatal morbidity” occurred in 54 (55.7%) of the 98 cases. “Postnatal mortality or severe neonatal morbidity” occurred in 32 (42.7%) cases, and “severe neonatal morbidity” was reported in 28 (37.3%) cases of 76. One infant was transferred to an external hospital, and detailed information about the status is missing except that it was discharged alive (Table 1). Of the mothers, 37 (37.8%) were hypertensive during their pregnancy, of whom 18 (18.4%) developed pre-eclampsia or hemolysis, elevated liver enzymes low platelets (HELLP) syndrome. CPR dropped below the threshold of one, signifying cerebral redistribution, in 73 (79.3%) cases and showed a median of 0.62 (25th/75th percentile: 0.47/0.86) before delivery. The median gestational age when CPR dropped below the threshold of 1 was 24 weeks (25th/75th percentile: 22.5/28) (Table 1).

**Table 1:** Maternal characteristics and neonatal outcomes.

** **	Median (25th/75th percentile)/*n* (%)	*n* ^a^
Maternal characteristics		* *
Maternal age (years)	29 (26/34)	98
Maternal BMI^b^ (before pregnancy) (kg/m^2^)	23.93 (20.86/28.99)	88
Hypertension (including preexisting)	37 (37.8)	98
Pre-eclampsia	18 (18.4)	98
Mean PI^d^ Aa. uterinae at 19 + 0 to 22 + 6 weeks of gestation	1.91 (1.3/2.27)	31
CPR^e^ < 1 during pregnancy	73 (79.3)	92
Gestational age when CPR < 1 was diagnosed (weeks)	24 (22.5/28)	73
CPR before delivery^f^ (up to 14 days)	0.62 (0.47/0.86)	79
AEDF UA^g^	50 (52.1)	96
REDF UA^h^	31 (32.3)	96
Ductus venosus reverse A-wave^i^	5 (9.8)	51
PETN^c^ intake of 50 mg twice daily	39 (39.8)	98
Pregnancy outcome		
Gestational age at birth (days)	188.5 (172/210.75)	98
Preterm birth before 28 + 0 weeks of gestation	57 (58.16)	98
Preterm birth before 28 + 0 weeks of gestation of life-born infants	37 (48.7)	76
Birth weight (g)	549 (427.5/870)	98
Birth weight (pc)	3 (1/6)	98
Birth length (cm)	32 (29/35)	63
Birth length (pc)	6 (2/9)	63
Head circumference (cm)	25 (22/26)	59
Head circumference (pc)	5 (3/13)	59
Male	62 (63.3)	98
Intrauterine death	22 (22.4)	98
Live births	76 (77.6)	98
Death (any, including pre- and postnatal cases)	42 (42.9)	98
Mode of delivery^j^		76
Vaginal	5 (6.6)	
Secondary C-section	11 (14.5)	
Primary C-section	60 (78.9)	
Emergency C-section^k^	15 (21.1%)	71
Delivery from 4.30 pm to 7.30 am	32 (40.1)	76
pH AU^l^ (per 1/10 unit)	7.28 (7.2/7.32)	62
APGAR score 5'	7 (5.75/8)	70
Fetal mortality or severe neonatal morbidity^m^	54 (55.7)	97
Neonatal outcome (of 76 live births)		
Postnatal mortality or severe neonatal morbidity^n^	32 (42.7)	75
Severe neonatal morbidity^o^	28 (37.3)	75
Postnatal death (before discharge)	20 (26.3)	76
Discharged alive	56 (73.7)	76
IVH (any)	24 (32)	75
Severe IVH^p^	7 (9.3)	75
PHT^q^ (any)	16 (21.3)	75
PHT requiring NO therapy	12 (16)	75
Gastrointestinal complications requiring surgery	15 (20)	75
BPD^r^	21 (29.2)	72
ROP^s^	14 (26)	54
Length of stay in NICU to discharge (of 56 survivors) (days)	79 (49/102)	55

^a^ Number of cases with available information.

^b^ Body mass index.

^c^ Pentaerythrityl tetranitrate.

^d^ Pulsatility index.

^e^ Cerebroplacental ratio: PI ACM/PI umbilical artery.

^f^ Values included if obtained within 14 days before delivery.

^g^ Absent end-diastolic flow in the umbilical arteries.

^h^ reverse end-diastolic flow in the umbilical arteries.

^i^ Reverse flow in the A-wave of the Doppler curve of the ductus venosus.

^j^ Vaginal includes cases of forceps and vacuum extraction; primary cesarean section is defined as performed before and secondary as performed following onset of labor.

^k^ Emergency cesarean section in Germany is defined as an acute emergency where delivery is required within 20 min.

^l^ Umbilical artery.

^m^ Any death or severe neonatal morbidity.

^n^ Postnatal death or severe neonatal morbidity.

^o^ IVH grade III or III+ and/or gastrointestinal complications requiring surgery and/or pulmonary hypertension requiring NO therapy.

^p^ Severe IVH means IVH III or IVH III+.

^q^ Pulmonary hypertension.

^r^ Bronchopulmonary dysplasia.

^s^ Retinopathy of prematurity.

Data on a group comparison of maternal characteristics and neonatal outcome data of 39 mothers who took PETN during pregnancy and 59 who did not are presented in Table 2. Mothers receiving PETN were older [median (25th/75th percentile): 31 (28/34) vs. 28 (25/33) years; *p* = 0.012] and had a higher median body mass index (BMI) [24.5 (21.9/31.8) vs. 22.6 (20.3/28.0); *p* = 0.047]. The number of cases with placental insufficiency characterized by impaired uteroplacental perfusion detected by Doppler was significantly higher in the PETN group (Table 2). Fetal brain sparing was diagnosed in 37 (94.9%) of the 39 mothers taking PETN compared to 36 of 59 mothers (67.9%) not taking PETN (*p* = 0.002). The median time point at which a CPR drop below the threshold of 1 was diagnosed was 23 (21.5/25) weeks in the PETN group compared to 26 (24/28) weeks (*p* = 0.001), and an absent or reversed flow in the umbilical artery was observed in 48.7% of those in the PETN group compared to 21.1% (*p* = 0.007). The median birth weight percentile was 2 (1/4) in the PETN group compared to 4 (1/7) in the no PETN group (*p* = 0.037). Regarding neonatal outcome parameters, there were no significant differences reported between the two groups (Table 2).

**Table 2:** Comparison of group characteristics according to PETN intake during pregnancy.

	Without PETN		With PETN		*p*
	Median (25th/75th)/*n* (%)		Median (25th/75th)/*n* (%)		
		*n* ^a^		*n* ^a^	
Maternal characteristics					
Maternal age (years)	28 (25/33)	59	31 (28/34)	39	0.012
Maternal BMI^b^ (before pregnancy) (kg/m^2^)	22.6 (20.31/27.99)	51	24.51 (21.93/31.75)	37	0.047
Hypertension (including preexisting)	22 (37.3)	59	15 (38.5)	39	1.000
Pre-eclampsia	12 (20.3)	59	6 (15.4)	39	0.603
Mean PI^c^ Aa. uterinae at 19 + 0 to 22 + 6	1.3 (0.74/1.91)	11	2.08 (1.87/2.32)	20	0.021
CPR < 1 in pregnancy (yes/no)	36 (67.9)	53	37 (94.9)	39	0.002
Gestational age when CPR was first < 1 (weeks)	26 (24/28)	36	23 (21.5/25)	37	0.001
CPR^d^ before delivery^e^ (up to 14 days)	0.66 (0.48/0.95)	42	0.61 (0.4/0.78)	37	0.280
AEDF UA^f^	24 (42.1)	57	26 (66.7)	39	0.023
REDF UA^g^	12 (21.1)	57	19 (48.7)	39	0.007
Ductus venosus reverse A-wave^h^	2 (7.7)	26	3 (12)	25	0.668
Duration of PETN^i^ intake (median) (days)			17 (7/44)	39	
Gestational age when PETN was started (weeks)			23 (21–25)	39	
Pregnancy outcome					
Gestational age at birth (days)	191 (171/216)	59	187 (172/209)	39	0.658
Preterm birth before 28 weeks	32 (54.2)	59	25 (64.1)	39	0.405
Preterm birth before 28 weeks of life-born infants	22 (46.8)	47	15 (51.7)	29	0.814
Birth weight (g)	590 (460/870)	59	495 (390/870)	39	0.284
Birth weight (pc)	4 (1/7)	59	2 (1/4)	39	0.037
Birth length (cm)	32 (29/36)	37	31 (28.62/34)	26	0.555
Birth length (pc)	6 (2/9)	37	5 (3/9)	26	0.459
Head circumference (cm)	25 (22/26)	34	23 (22/26)	25	0.195
Head circumference (pc)	5 (4/13)	34	5 (3/12)	25	0.602
Male	36 (61)	59	26 (66.7)	39	0.670
Death (any, including pre- and postnatal cases)	22 (37.3)	59	20 (51.3)	39	0.212
Mode of delivery^j^		47		29	0.059
Vaginal	5 (10.6)		0 (0)		
Secondary C-section	8 (17)		3 (10.3)		
Primary C-section	34 (72.3)		26 (89.7)		
Emergency C-section^k^	8 (17)	47	7 (25)	28	0.407
Delivery 4.30 pm to 7.30 am	21 (44.7)	47	11 (37.9)	29	0.565
pH AU^l^ (per 1/10 unit)	7.29 (7.22/7.32)	38	7.24 (7.18/7.29)	24	0.023
APGAR score 5'	7 (5.75/8)	42	7 (5.25/8)	28	0.995
Neonatal outcome (of 76 live births)					
Intrauterine death	12 (20.3)	59	10 (25.6)	39	0.623
Live births	47 (79.7)	59	29 (74.4)	39	0.623
Mortality or severe neonatal morbidity^m^	29 (49.2)	59	25 (65.8)	38	0.143
Postnatal mortality and severe neonatal morbidity^n^	17 (36.2)	47	15 (53.6)	28	0.156
Severe neonatal morbidity^o^	14 (29.8)	47	14 (50)	28	0.091
Postnatal death (before discharge)	10 (21.3)	47	10 (34.5)	29	0.284
Discharged alive	37 (78.7)	47	19 (65.5)	29	0.284
IVH (any)	15 (31.9)	47	9 (32.1)	28	1.000
Severe IVH^p^	4 (8.5)	47	3 (10.7)	28	1.000
PHT^q^ (any)	8 (17)	47	8 (28.6)	28	0.258
PHT requiring NO therapy	5 (10.6)	47	7 (25)	28	0.116
Gastrointestinal complications requiring surgery	8 (17)	47	7 (25)	28	0.552
BPD^r^	14 (31.1)	45	7 (25.9)	27	0.790
ROP^s^	10 (27.8)	36	4 (22.2)	18	0.489
Length of stay in NICU (survivors) (days)	79 (49.5/104)	37	77 (46/96.75)	18	0.713

^a^ Number of cases with available information.

^b^ Body mass index.

^c^ Pulsatility index.

^d^ Cerebroplacental ratio: PI ACM/PI umbilical artery.

^e^ Values included if obtained within 14 days before delivery.

^f^ Absent end-diastolic flow in the umbilical arteries.

^g^ Reverse end-diastolic flow in the umbilical arteries.

^h^ Reverse flow in the A-wave of the Doppler curve of the ductus venosus.

^i^ Pentaerythrityl tetranitrate.

^j^ Vaginal includes cases of forceps and vacuum extraction; primary cesarean section is defined as performed before and secondary as performed following the onset of labor.

^k^ Emergency cesarean section in Germany is defined as an acute emergency where delivery is required within 20 min.

^l^ Umbilical artery.

^m^ Any death, including pre- and postnatal cases, or severe neonatal morbidity.

^n^ Postnatal death or severe neonatal morbidity.

^o^ IVH grade III or III+ and/or gastrointestinal complications requiring surgery and/or pulmonary hypertension requiring NO therapy.

^p^ Severe IVH means IVH III or IVH III+.

^q^ Pulmonary hypertension.

^r^ Bronchopulmonary dysplasia.

^s^ Retinopathy of prematurity.

Using univariable analysis, we tested the influence of different risk factors on neonatal outcome parameters. Significant results are reported in Table 3. The gestational age when CPR dropped below the threshold of 1 significantly impacted all considered outcomes except IVH (Table 3).

**Table 3:** Univariable analysis of the impact of different risk factors on neonatal outcome.^a^

Neonatal outcome	Risk factor	OR	95% CI	*p*
Live birth	GA^b^ at birth (days)	1.053	1.022 to 1.085	0.001
	Birth weight (pc)	1.588	1.213 to 2.079	0.001
	GA CPR < 1 (weeks)	1.654	1.197 to 2.285	0.002
	DV^c^: reverse A-wave	0.047	0.005 to 0.395	0.005
Death (any)	GA at birth (days)	0.945	0.922 to 0.969	<0.001
	Birthweight (pc)	0.693	0.580 to 0.829	<0.001
	GA CPR^d^ < 1 (weeks)	0.621	0.487 to 0.792	<0.001
Postnatal death	GA at birth (days)	0.974	0.949 to 0.999	0.043
Mortality or severe neonatal morbidity^e^	GA at birth (days)	0.941	0.917 to 0.965	<0.001
	Birthweight (pc)	0.668	0.561 to 0.797	<0.001
	GA CPR < 1 (weeks)	0.577	0.447 to 0.745	<0.001
	APGAR score 5’	0.714	0.529 to 0.962	0.027
Postnatal mortality or Severe neonatal morbidity^f^	GA at birth (days)	0.948	0.921 to 0.975	0.000
	Birthweight (pc)	0.706	0.578 to 0.863	0.001
	GA CPR < 1 (weeks)	0.632	0.487 to 0.820	0.001
	APGAR score 5’	0.714	0.529 to 0.962	0.027
	High-risk obstetric history^g^ (yes)	3.195	1.217 to 8.392	0.018
Severe neonatal morbidity^h^	GA at birth (days)	0.952	0.926 to 0.979	0.001
	Birthweight (pc)	0.718	0.586 to 0.880	0.001
	GA CPR < 1 (weeks)	0.591	0.442 to 0.788	<0.001
	pH AU^i^ (per 1/10 unit)	<0.001	0.000 to 0.191	0.016
IVH (any)	Maternal age (years)	0.908	0.831 to 0.991	0.032
Severe IVH^j^	Emergency C-section	6.909	1.353 to 35.269	0.020
PHT^k^ (any)	Birthweight (pc)	0.756	0.598 to 0.956	0.020
	GA CPR < 1 (weeks)	0.670	0.503 to 0.893	0.006
PHT requiring NO therapy	Birthweight (pc)	0.767	0.590 to 0.996	0.047
	GA CPR < 1 (weeks)	0.711	0.535 to 0.945	0.019
Gastrointestinal complications requiring surgery	GA at birth (days)	0.949	0.917 to 0.983	0.003
	Birthweight (pc)	0.779	0.616 to 0.985	0.037
	GA CPR < 1 (weeks)	0.591	0.411 to 0.848	0.004
	Mode of delivery^l^			0.017
	Secondary vs. primary C-section	7.600	1.884 to 31.067	0.004
	Vaginal vs. primary C-section	1.594	0.157 to 16.130	0.693
BPD^m^	GA at birth (days)	0.943	0.912 to 0.974	<0.001
	GA CPR < 1 (weeks)	0.724	0.563 to 0.931	0.012
ROP^n^	GA at birth (days)	0.913	0.870 to 0.957	<0.001
	APGAR score at 5 min	0.444	0.250 to 0.788	0.006
Length of stay in NICU to discharge (survivors)	GA at birth (days)	−1.385	−1.633 to 1.138	<0.001
	GA CPR < 1 (weeks)	−7.162	−9.622 to 4.701	<0.001
	DV: reverse A-wave	70.467	11.668 to 129.265	0.021
	Mode of delivery			0.003
	Secondary vs. primary C-section	27.34	3.101 to 51.578	0.028
	Vaginal vs. primary C-section	52.911	17.339 to 88.483	0.004
	High-risk obstetric history (yes)	21.296	3.947 to 38.644	0.017
	APGAR score 5'	−13.229	−18.072 to 8.387	<0.001

^a^ Only significant results are displayed.

^b^ Gestational age.

^c^ Reverse flow in the A-wave of the Doppler curve of the ductus venosus.

^d^ Cerebroplacental ratio.

^e^ Any death, including pre- and postnatal cases, or severe neonatal morbidity.

^f^ Postnatal death or severe neonatal morbidity

^g^ High-risk obstetric history including at least one of the following: intrauterine death, abortion, stillbirth, or placental abruption.

^h^ IVH grade III or III+ and/or gastrointestinal complications requiring surgery and/or pulmonary hypertension requiring NO therapy.

^i^ Umbilical artery.

^j^ IVH grade III or III+.

^k^ Pulmonary hypertension.

^l^ Vaginal includes cases of forceps and vacuum extraction; primary cesarean section is defined as performed before and secondary as performed following the onset of labor.

^m^ Bronchopulmonary dysplasia.

^n^ Retinopathy of prematurity.

**Figure 2:**
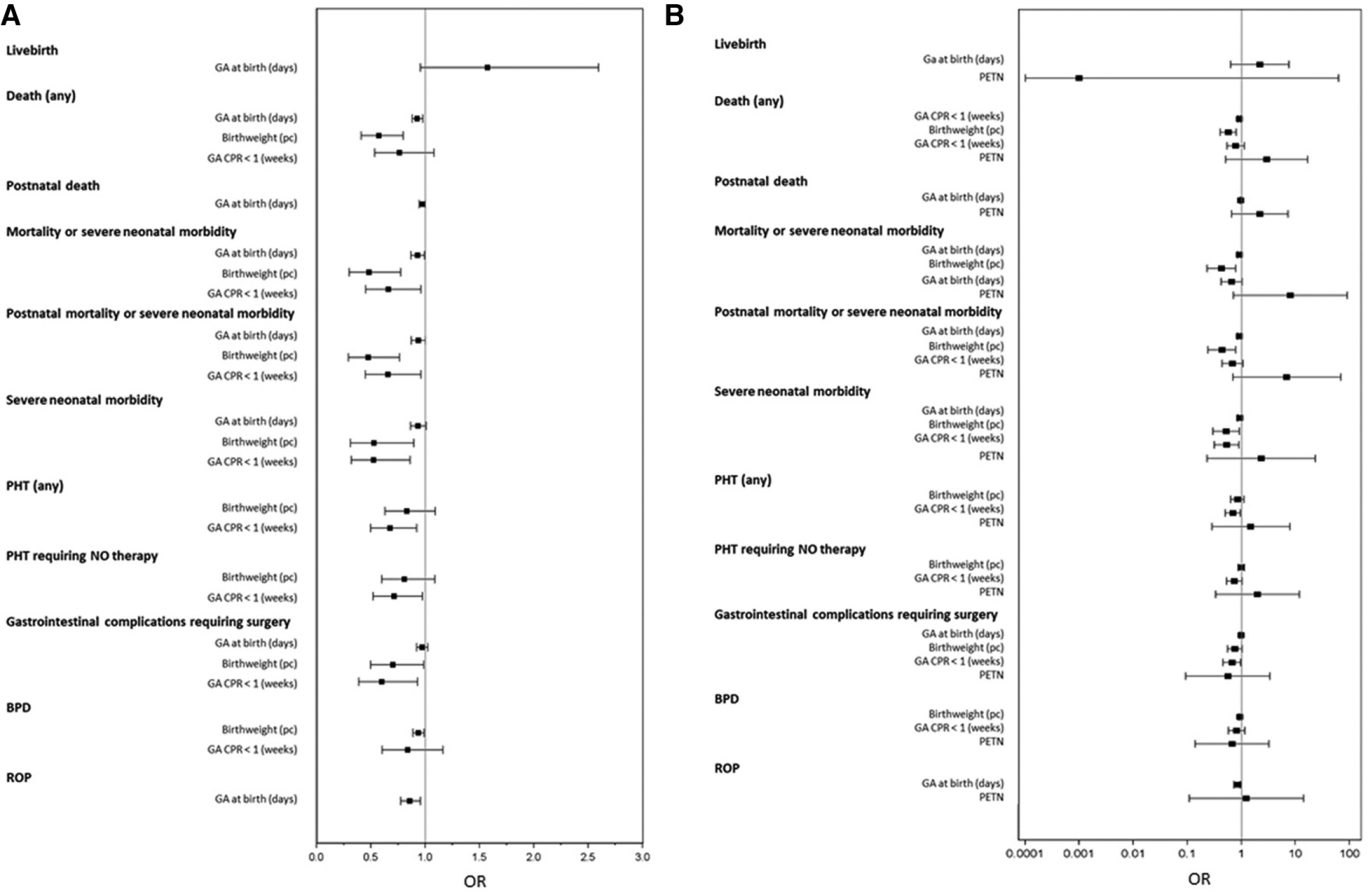
Forest plot of adjusted OR for neonatal outcome. (**A**) Adjustment was made for significantly impacting characteristics revealed from the unadjusted analysis (Table 3), for newborn sex, maternal age, and maternal BMI, and (**B**) additionally for PETN. Results are displayed for the characteristics such as gestational age at birth, birth weight percentile, gestational age when CPR dropped below the threshold of 1, and PETN in (**B**).

Subsequently, we tested the impact of significant risk factors on neonatal outcomes in a multivariable analysis. Each outcome parameter was adjusted for significant risk factors identified in the univariable analysis. An additional adjustment was made for the sex of the newborn, maternal age, and BMI. Furthermore, we performed a multivariable analysis to test for the impact of PETN. The complete results are displayed in Supplementary Table S1. In Table 4 and Figure 2, we show the results for the adjusted effects of gestational age at birth, birth weight percentile, gestational age at which the drop in CPR below the threshold was detected, and PETN intake (Table 4 and Figure 2).

**Table 4:** Multivariable analysis of gestational age at birth, birth weight percentile, gestational age when CPR indicates fetal central redistribution, and PETN intake on neonatal outcome.

Outcome	Risk factor	Multivariable analysis^a^			Multivariable analysis including PETN^b^		
		OR	95% CI	*p*	OR	95% CI	*p*
Live birth^c^	GA^d^ at birth (days)	1.57	0.954 to 2.594	0.076	2.19	0.628 to 7.603	0.219
	Birth weight (pc)	NA			NA		
	GA CPR < 1 (weeks)	NA			NA		
	PETN	NA			0.00	0.000 to 62.727	0.199
Death (any)	GA at birth (days)	0.93	0.880 to 0.977	**0**.**005**	0.92	0.865 to 0.973	**0**.**004**
	Birth weight (pc)	0.57	0.412 to 0.797	**0**.**001**	0.57	0.406 to 0.801	**0**.**001**
	GA CPR < 1 (weeks)	0.76	0.537 to 1.080	0.127	0.78	0.545 to 1.127	0.189
	PETN		NA		2.91	0.508 to 16.675	0.230
Postnatal death	GA at birth (days)	0.97	0.946 to 1.000	0.052	0.97	0.947 to 1.002	0.069
	Birth weight (pc)	NA			NA		
	GA CPR < 1 (weeks)	NA			NA		
	PETN	NA			2.19	0.662 to 7.280	0.199
Mortality or severe neonatal morbidity^e^	GA at birth (days)	0.93	0.867 to 0.995	**0**.**034**	0.91	0.843 to 0.987	**0**.**023**
	Birth weight (pc)	0.48	0.301 to 0.773	**0**.**002**	0.43	0.233 to 0.786	**0**.**006**
	GA CPR < 1 (weeks)	0.66	0.454 to 0.960	**0**.**030**	0.66	0.420 to 1.033	*0*.*069*
	PETN	NA			8.03	0.717 to 89.924	0.091
Postnatal mortality or severe neonatal morbidity^f^	GA at birth (days)	0.94	0.874 to 1.002	0.057	0.92	0.851 to 0.997	***0***.***042***
	Birth weight (pc)	0.47	0.294 to 0.764	**0**.**002**	0.43	0.240 to 0.787	**0**.**006**
	GA CPR < 1 (weeks)	0.65	0.447 to 0.961	**0**.**031**	0.68	0.437 to 1.068	*0*.*095*
	PETN	NA			6.934	0.692 to 69.489	0.100
Severe neonatal morbidity^g^	GA at birth (days)	0.93	0.863 to 1.007	0.076	0.93	0.852 to 1.007	0.073
	Birth weight (pc)	0.53	0.313 to 0.896	**0**.**018**	0.52	0.295 to 0.917	**0**.**024**
	GA CPR < 1 (weeks)	0.52	0.320 to 0.862	**0**.**011**	0.53	0.317 to 0.898	**0**.**018**
	PETN	NA			2.32	0.231 to 23.357	0.474
IVH^h^ (any)	GA at birth (days)	NA			NA		
	Birth weight (pc)	NA			NA		
	GA CPR < 1 (weeks)	NA			NA		
	PETN	NA			1.33	0.427 to 4.145	0.623
Severe IVH	GA at birth (days)	NA			NA		
	Birth weight (pc)	NA			NA		
	GA CPR < 1 (weeks)	NA			NA		
	PETN	NA			1.75	0.255 to 12.021	0.569
PHT^i^ (any)	GA at birth (days)	NA			NA		
	Birth weight (pc)	0.83	0.631 to 1.093	0.185	0.84	0.635 to 1.114	0.228
	GA CPR < 1 (weeks)	0.68	0.499 to 0.920	**0**.**013**	0.69	0.504 to 0.950	**0**.**023**
	PETN	NA			1.500	0.285 to 7.902	0.633
PHT requiring NO therapy	GA at birth (days)	NA			NA		
	Birth weight (pc)	0.80	0.599 to 1.089	0.161	0.99	0.873 to 1.135	0.941
	GA CPR < 1 (weeks)	0.71	0.521 to 0.975	**0**.**034**	0.74	0.536 to 1.026	0.071
	PETN	NA			1.99	0.334 to 11.871	0.450
Gastrointestinal complications requiring surgery	GA at birth (days)	0.97	0.921 to 1.025	0.296	0.98	0.937 to 1.034	0.534
	Birth weight (pc)	0.70	0.499 to 0.986	**0**.**041**	0.75	0.552 to 1.024	*0*.*070*
	GA CPR < 1 (weeks)	0.60	0.388 to 0.931	**0**.**023**	0.67	0.457 to 0.980	**0**.**039**
	PETN	NA			0.56	0.093 to 3.377	0.528
BPD^j^	GA at birth (days)	0.94	0.889 to 0.989	**0**.**017**	0.94	0.890 to 0.991	**0**.**022**
	Birth weight (pc)	NA			NA		
	GA CPR < 1 (weeks)	0.84	0.602 to 1.165	0.292	0.81	0.571 to 1.159	0.253
	PETN	NA			0.67	0.139 to 3.236	0.618
ROP^k^	GA at birth (days)	0.86	0.773 to 0.954	**0**.**005**	0.86	0.722 to 0.954	**0**.**005**
	Birth weight (pc)	NA			NA		
	GA CPR < 1 (weeks)	NA			NA		
	PETN	NA			1.24	0.108 to 14.117	***0***.***864***
Length of stay in NICU—discharge (survivors)	GA at birth (days)	−0.79	−1.547 to 0.037	**0**.**041**	−0.48	−1.153 to 0.180	0.139
	Birth weight (pc)	NA			NA		
	GA CPR < 1 (weeks)	−3.63	−7.024 to 0.235	**0**.**038**	−4.09	−6.914 to 1.267	**0**.**008**
	PETN	NA			−21.14	−37.429 to 4.853	**0**.**015**

Complete results of the multivariable analysis are displayed in Supplementary Table S1. Results in bold indicate significance. Outcomes and risk factors printed in italics indicate a change in significance upon adjustment for PETN.

^a^ Adjustment was made for significant results retrieved from the univariate analysis (Table 3) and for newborn sex, maternal age, and BMI.

^b^ Additional adjustment was made for PETN.

^c^ For live birth, due to rare events we applied backward stepwise logistic regression. Birth weight (pc), GA CPR < 1, and DV: reverse A-wave were not included in the final model.

^d^ Gestational age.

^e^ IVH grade III or IV, gastrointestinal complications requiring surgery, pulmonary hypertension requiring NO therapy, and/or the occurrence of death (any) before discharge from the hospital.

^f^ IVH grade III or IV, gastrointestinal complications requiring surgery, pulmonary hypertension requiring NO therapy, and/or the occurrence of death after birth before discharge from the hospital in all live births.

^g^ IVH grade III or IV and/or gastrointestinal complications requiring surgery and/or pulmonary hypertension requiring NO therapy in all surviving newborns.

^h^ Intraventricular hemorrhage.

^i^ Pulmonary hypertension.

^j^ Bronchopulmonary dysplasia.

^k^ Retinopathy of prematurity.

The multivariable analysis revealed that gestational age at birth still significantly impacted any death, “mortality or severe neonatal morbidity,” BPD, and the length of stay in the NICU. The odds of any death decreased per achieved day of pregnancy length with an odds ratio (OR) of 0.927 (95% CI: 0.880–0.977) and for the composite outcome of “mortality or severe neonatal morbidity” with an OR of 0.929 (95% CI: 0.867–0.995) (Table 4 and Figure 2).

The birth weight percentile significantly influenced any death, “mortality or severe neonatal morbidity,” “postnatal death or severe neonatal morbidity,” “severe neonatal morbidity,” and the occurrence of gastrointestinal complications requiring surgery. Per additionally gained birthweight percentile, the odds of the following was decreased: any death (OR 0.573; 95% CI: 0.412–0.797); the composite outcome of “mortality or severe neonatal morbidity” (OR 0.482; 95% CI: 0.301–0.773); “postnatal death or severe neonatal morbidity” (OR 0.474; 95% CI: 0.294–0.764); and for the surviving neonates, the odds of “severe neonatal morbidity” (OR 0.529; 95% CI: 0.313–0.896) (Table 4 and Figure 2).

Gestational age, when a drop in CPR below the threshold of 1 was first diagnosed, significantly affected all composite outcome parameters, as well as the occurrence of PHT, PHT requiring NO therapy, gastrointestinal complications requiring surgery, and the length of stay in the NICU. For each week of pregnancy gained before the drop of CPR below 1, the odds of the composite outcome “mortality or severe neonatal morbidity” was reduced (OR 0.660; 95% CI: 0.454–0.960), “postnatal death or severe neonatal morbidity” was reduced (OR 0.655; 95% CI: 0.447–0.961); and “severe neonatal morbidity” was reduced (OR 0.525; 95% CI: 0.320–0.862) (Table 4 and Figure 2).

In the multivariable analysis including PETN intake, PETN was not shown to independently impact the reported outcome parameters except for the length of stay in the NICU, which was significantly reduced by a mean of 21 days (Table 4 and Figure 2).

## 4 Discussion

Our data, retrieved from a retrospective cohort analysis of 98 cases of FGR neonates born early preterm, confirm the tremendous impact of both gestational age at birth and birth weight percentile on neonatal outcome. In our cohort, the odds of developing any of the chosen composite outcome parameters or of mortality was reduced by nearly 8% per prolonged day of pregnancy and nearly halved with each achieved percentile in birth weight (Table 4).

Furthermore, the early occurrence of fetal brain sparing, indicated by a drop in CPR below the threshold, significantly impacted neonatal outcomes. In our cohort, we observed a reduction in the odds of developing any of the chosen composite outcome parameters by approximately 40% with each achieved week of gestational age before brain sparing was detected. In addition, the odds of developing PHT or a PHT requiring NO therapy and gastrointestinal complications requiring surgery was reduced by 30% and 40%, respectively, with each achieved week of gestation before CPR dropping below 1 (Table 4).

Within our study cohort of 98 mothers, 39 received PETN during pregnancy. A comparison of group characteristics between mothers who received PETN and those who did not are displayed in Table 2, demonstrating that mothers who received PETN were older and had a higher BMI. Impaired fetoplacental and uteroplacental perfusion was diagnosed more frequently in the PETN cases, reflecting that PETN was given predominantly in cases with a high risk of impaired fetal development and in which alterations in fetal perfusion were detected at an earlier gestational age and were more severe (Table 2). Taking this into account, it is remarkable that no difference in neonatal outcome was observed between the groups (Table 2).

A multivariable analysis including PETN revealed that PETN intake reduced the length of stay in the NICU by approximately 21 days; however, we could not demonstrate an independent influence of PETN on the chosen composite outcome parameters. Interestingly, upon adjusting for PETN intake, gestational age when fetal brain sparing was diagnosed no longer impacted the composite outcomes of “mortality or severe neonatal morbidity” and “postnatal death or severe neonatal morbidity” and the development of any PHT (Figure 2). Although the data must be interpreted with caution, they suggest an effect of PETN, predominantly in cases of altered fetoplacental perfusion. This concurs with our results from the PETN pilot study (10). However, the number of included cases in our study is low, the study is of a retrospective nature, and the confidence intervals retrieved by the multivariable analysis are partially large (Figure 2).

The mortality rates in our cohort correlate with otherwise reported frequencies of 22.4% prenatally, 26.3% postnatally, and 42.9% for any death. In a comprehensive review of the outcome of extremely premature infants, summarizing the data from the last two decades of the last century, the authors report mortality rates in the range of 30%–50% (17). In a more recent review reporting the data of two large multicenter trials, 78% of extremely preterm-born children survived into childhood (18). Mortality rates of growth-restricted neonates born preterm can be retrieved from the STRIDER study, which included pregnant women with a growth-restricted fetus at weeks 26–30 who then received either sildenafil or placebo (19). The reported perinatal mortality was 15%–30%, which is consistent with our data (20, 21).

The risk of developing severe morbidity in early preterm infants is summarized by Glass et al. as being 63.7 for any ROP, 14.1 for severe IVH, 10.1 for surgical NEC, and approximately 40 for BPD (17), also in line with the data observed in this study (Table 1). The frequencies in the STRIDER studies, which analyzed a growth-restricted preterm cohort corresponding to our cohort, were reported to be 17% for ROP, 26% for IVH (any), 21% for NEC, and 80% for composite perinatal adverse outcomes (defined as perinatal death or neonatal morbidity) (21). The rates observed in our study were markedly lower (Table 1). This could be explained by the demonstrated impact of gestational age at birth and birth weight centile, both of which were lower in the STRIDER cohorts (19–21).

Data on infants born with very low birth weight (<1,500 g) describe frequencies in the range of 20%–25% for IVH (22). The higher incidence of 32% in our cohort can be explained by the increased IVH risk in growth-restricted neonates. The reported prevalence of PHT in preterm neonates is 23%–37% (23). In our cohort of early preterm growth-restricted neonates, the observed rates of 21% for PHT and 16% for PHT requiring NO therapy are comparably low (Table 4 and Figure 2).

In addition to the known influence of gestational age and birth weight percentile on neonatal outcome, this study revealed a profound impact of gestational age on mortality and severe neonatal morbidity when fetal brain sparing occurs (Table 4 and Figure 2). With each additional week of gestation gained before fetal brain sparing was observed, the probability of severe neonatal morbidity decreased by approximately 40% (combined outcome parameters), and of the development of PHT, or gastrointestinal complications requiring surgery, decreased by 30% and 40%, respectively (Table 4). This coincides with published data that states that as gestational age increases, the overall outcome improves. However, the impact of gestational age when fetal brain sparing was detected was still significant after the adjustment for gestational age at birth (Table 4).

Studies investigating the impact of CPR values on outcomes in growth-restricted preterm infants with gestational age at birth below 32 weeks are few. Summarizing data from 128 studies and a total of 47,748 pregnancies, CPR determined before delivery has been shown to be predictive for adverse perinatal outcomes (24). However, the study population was heterogeneous, including growth-restricted and appropriate-grown neonates and preterm birth was defined as birth before 37 weeks of gestation. The review by DeVore et al. summarized data on CPR differentiating between small for gestational age (SGA) and appropriate for gestational age (AGA) born children, as well as between early-onset SGA (before 34 weeks) and late-onset SGA (34 weeks and older) (7). The authors demonstrated that in early-onset SGA with abnormal CPR, gestational age at birth, mean birth weight, and mean birth weight centile were lower, and frequencies of cesarean delivery due to fetal distress, APGAR scores at 5 min of less than 7 points, neonatal acidosis, admission to the NICU, and perinatal death were higher (7). The PORTO study, a prospective observational study investigating a large case series of 881 growth-restricted fetuses, found a cutoff of 1.0 for CPR to be predictive of an unfavorable perinatal outcome with a specificity of 85% and a sensitivity of 66% (8). The mean gestational age at birth was 37.7 weeks, and the mean gestational age at detection of abnormal CPR was 33 weeks (25th/75th percentile: 28.7/35.9). In accordance, we set the cutoff for defining an abnormal CPR in our cohort as 1.0. However, different cutoff values and percentiles for CPR according to gestational age have been published (25). It has been discussed that gestational age-specific cutoff values are not superior to categorical cutoffs (26). Since our study includes a heterogeneous cohort and our data were retrieved over a long period, we decided to use a threshold of 1 to define pathological CPR, as this was used as the clinical cutoff during the time the study participants were managed.

Recently, a number of studies investigating CPR in term infants demonstrated a predictive value of CPR below the 5th percentile for adverse perinatal outcomes to be favorable. These data from AGA uncomplicated pregnancies demonstrate an association between a preterm drop in CPR and perinatal outcome (25, 27). It is not only the drop in CPR below the cutoff that corresponds to the perinatal outcome but also the actual. As the CPR decreases, the severity of neonatal morbidity increases (28). In a recent study presenting the secondary analysis of the PORTO trial, growth-restricted pregnancies with a CPR < 1 had a significantly increased risk of delayed neurodevelopment at 3 years of age when compared with pregnancies with abnormal UA-PI alone. These data strongly confirm our observation that fetal brain sparing predicts adverse outcomes in FGR fetuses (29). To our knowledge, there has been no study investigating the impact of the first detection of fetal brain sparing. Our results indicate that upcoming studies should emphasize this distinction.

To summarize, there is profound evidence that CPR below the threshold is associated with an adverse perinatal outcome. In our cohort, the disadvantage of being diagnosed with brain sparing was not as evident, which could be assigned to the PETN treatment, although the evidence is weak and needs to be confirmed in further studies. However, some support for this assumption can be driven by data retrieved from the PETN pilot study, demonstrating a later gestational age before CPR dropped below the threshold in the PETN cohort (11).

A strength of our study is the monocentric setting, which ensured comparable postnatal treatment in the cases evaluated. Furthermore, the data quality is good, providing complete data sets for the majority of the cases meeting the inclusion criteria. Nevertheless, our study has all the disadvantages of being a retrospective study. Moreover, the effect of PETN in pregnancies with early-onset FGR can only be of a descriptive nature, since the reasons for deciding on PETN treatment were particularly heterogeneous and PETN treatment was predominantly offered to severe cases and not to all women on a regular basis.

In conclusion, we provide data on the significant impact of not only gestational age and birth weight percentile on the immediate outcome of very preterm growth-restricted newborns but also demonstrate a significant impact of the time point at which fetal brain sparing is first diagnosed during pregnancy. This finding will enable obstetricians to further differentiate between fetuses at risk when managing pregnancies complicated by growth restrictions. PETN intake did reduce the impact of early brain sparing on postnatal death and might have the potential to mitigate adverse outcomes in severe cases of early FGR.

## Data Availability

The original contributions presented in the study are included in the article/**Supplementary Material**, further inquiries can be directed to the corresponding author.
