# Supplementary material for: Gestational age at birth, birth weight, and gestational age when intrauterine brain sparing occurs determines the neonatal outcome in growth-restricted infants born before 32 weeks of gestation: a retrospective cohort analysis
Source: Front Pediatr. 2024 Jul 4;12:1377982. doi: 10.3389/fped.2024.1377982 (PMC11254702; doi:10.3389/fped.2024.1377982)
Supplement: Supplementary file 1 [file Table1.docx]

Supplemental Table 1. Multivariate Analysis of prenatal influencers on neonatal outcome in infants born growth restricted before completed 32 weeks of gestation. Adjustment was done for significant results of the univariate analysis and in a second analysis additionally for PETN. Result are reported for significant results of the univariate analysis.

|  |  | **Adjustment was done for significant resuls from univariate analysis** | | | | **Adjustment was done for significant resuls from univariate analysis and PETN intake** | | |
| --- | --- | --- | --- | --- | --- | --- | --- | --- |
| **Neonatal Outcome** | **Risk factor** | **OR** | **confidence interval** | **p** | **OR** | | **confidence interval** | **p** |
| **Death (any)** | *GA at birth* (days) | *0.927* | *0.880 - 0.977* | ***0.005*** | 0.18 | | 0.865 - 0.973 | **0.004** |
|  | Birthweight (pc) | *0.573* | *0.412 - 0.797* | ***0.001*** | 0.570 | | 0.406 - 0.801 | **0.001** |
|  | GA CPR < 1(weeks) | 0.762 | 0.537 - 1.080 | 0.127 | 0.784 | | 0.545 - 1.127 | 0.189 |
|  | Maternal age (years) | 0.958 | 0.828 - 1.109 | 0.566 | 0.928 | | 0.796 - 1.083 | 0.344 |
|  | Maternal BMI (kg/m^2^) | 1.123 | 0.986 - 1.279 | 0.081 | 1.112 | | 0.975 - 1.268 | 0.114 |
|  | gender: female | 0.284 | 0.043 - 1.873 | 0.191 | 0.217 | | 0.028 - 1.680 | 0.143 |
|  | PETN |  |  |  | 2.911 | | 0.508 - 16.675 | 0.230 |
| **Postnatal death** | GA at birth (days) | 0.973 | 0.946 - 1.000 | 0.052 | 0.974 | | 0.947 - 1.002 | 0.069 |
|  | Maternal age (years) | 0.918 | 0.834 - 1.010 | 0.078 | 0.895 | | 0.803 - 0.997 | **0.045** |
|  | Maternal BMI (kg/m^2^) | 1.016 | 0.935 - 1.104 | 0.714 | 1.002 | | 0.919 - 1.092 | 0.968 |
|  | gender: female | 0.83 | 0.272 - 2.533 | 0.743 | 0.842 | | 0.272 - 2.603 | 0.765 |
|  | PETN |  |  |  | 2.195 | | 0.662 - 7.280 | 0.199 |
| **Mortality and severe neonatal morbidity** | *GA at birth* (days) | 0.929 | 0.867 - 0.995 | **0.034** | 0.912 | | 0.843 - 0.987 | **0.023** |
|  | Birthweight (pc) | 0.482 | 0.301 - 0.773 | **0.002** | 0.428 | | 0.233 - 0.786 | **0.006** |
|  | *GA CPR < 1*(weeks) | 0.660 | 0.454 - 0.960 | **0.030** | 0.659 | | 0.420 - 1.033 | 0.069* |
|  | APGAR score at 5 minutes | 0.885 | 0.450 - 1.743 | 0.725 | 0.729 | | 0.335 - 1.589 | 0.427 |
|  | Maternal age (years) | 1.067 | 0.923 - 1.234 | 0.381 | 1.019 | | 0.851 - 1.221 | 0.839 |
|  | Maternal BMI (kg/m^2^) | 1.071 | 0.916 - 1.252 | 0.392 | 1.027 | | 0.865 - 1.219 | 0.761 |
|  | gender: female | 0.105 | 0.005 - 2.362 | 0.156 | 0.033 | | 0.001 -2.140 | 0.109 |
|  | PETN |  |  |  | 8.032 | | 0.717 - 89.924 | 0.091 |
| **Postnatal mortality and severe neonatal morbidity** | *GA at birth* (days) | 0.936 | 0.874 - 1.002 | 0.057 | 0921 | | 0.851 - 0.997 | **0.042*** |
|  | Birthweight (pc) | 0.474 | 0.294 - 0.764 | **0.002** | 0.435 | | 0.240 - 0.787 | 0.006 |
|  | *GA CPR < 1*(weeks) | 0.655 | 0.447 - 0.961 | **0.031** | 0.683 | | 0.437 - 1.068 | 0.095* |
|  | APGAR score at 5 minutes | 0.910 | 0.453 - 1.86 | 0.790 | 2.660 | | 0.328 - 21.592 | 0.360 |
|  | Maternal age (years) | 1.034 | 0.884 - 1.208 | 0.678 | 0.777 | | 0.343 - 1.761 | 0.546 |
|  | Maternal BMI (kg/m^2^) | 1.081 | 0.915 - 1.278 | 0.359 | 0.991 | | 0.820 - 1.198 | 0.927 |
|  | Gender: female | 0.128 | 0.006 - 2.949 | 0.199 | 1.039 | | 0.864 - 1.249 | 0.683 |
|  | PETN |  |  |  | 0.530 | | 0.001 - 3.471 | 0.168 |
| **Severe neonatal morbidity** | GA at birth (days) | 0.932 | 0.863 - 1.007 | 0.076 | 6.934 | | 0.692 - 69.489 | 0.100 |
|  | Birthweight (pc) | *0.529* | *0.313 - 0.896* | ***0.018*** | 0.926 | | 0.852 - 1.007 | 0.073 |
|  | *GA CPR < 1* (weeks) | *0.525* | *0.320 - 0.862* | ***0.011*** | 0.521 | | 0.295 - 0.917 | **0.024** |
|  | NA pH | *0.682* | 0.230 - 2.023 | 0.490 | 0.533 | | 0.317 - 0.898 | **0.018** |
|  | Maternal age (years) | 1.127 | 0.938 - 1.354 | 0.203 | 0.770 | | 0.258 - 2.296 | 0.639 |
|  | Maternal BMI (kg/m^2^) | 1.074 | 0.889 - 1.297 | 0.462 | 1.107 | | 0.910 - 1.346 | 0.310 |
|  | gender: female | 0.075 | 0.002 - 3.661 | 0.192 | 1.064 | | 0.879 - 1.288 | 0.526 |
|  | PETN |  |  |  | 0.068 | | 0.001 - 4.602 | 0.211 |
| **IVH (any)** | *Maternal age* (years) | *0.91* | *0.832 - 0.996* | ***0.040*** | 2.325 | | 0.231 - 23.357 | 0.474 |
|  | Maternal BMI (kg/m^2^) | 0.963 | 0.884 - 1.050 | 0.392 | 0.904 | | 0.822 - 0.994 | **0.038** |
|  | gender: female | 1.373 | 0.470 - 4.011 | 0.562 | 0.960 | | 0.880 - 1.047 | 0.355 |
|  | PETN |  |  |  | 0.904 | | 0.822 - 0.994 | 0.551 |
| **severe IVH** (grade III or III+) | *Emergency C-section* | *7.352* | *1.222 - 44.24* | *0.029* | 1.330 | | 0.427 - 4.145 | 0.623 |
|  | Maternal age (years) | 0.909 | 0.774 - 1.066 | 0.240 | 6.842 | | 1.135 - 41.245 | **0.036** |
|  | Maternal BMI (kg/m^2^) | 3.996 | 0.795 - 1.104 | 0.645 | 0.895 | | 0.753 - 1.064 | 0.209 |
|  | gender: female | 0.985 | 0.170 - 5.696 | 0.987 | 0.925 | | 0.779 - 1.099 | 0.375 |
|  | PETN |  |  |  | 1.109 | | 0.186 - 6.611 | 0.910 |
| **PHT (any)** | Birthweight (pc) | 0.830 | 0.631 - 1.093 | 0.185 | 1.750 | | 0.255 - 12.021 | 0.569 |
|  | *GA CPR < 1* (weeks) | *0.677* | *0.499 - 0.920* | ***0.013*** | 0.841 | | 0.635 - 1.114 | 0.228 |
|  | Maternal age (years) | 0.909 | 0.791 - 1.045 | 0.179 | 0.692 | | 0.504 - 0.950 | **0.023** |
|  | Maternal BMI (kg/m^2^) | 0.977 | 0.860 - 1.109 | 0.715 | 0.898 | | 0.774 - 1.043 | 0.159 |
|  | gender: female | 0.782 | 0.154 - 3.962 | 0.766 | 0.971 | | 0.854 - 1.105 | 0.660 |
|  | PETN |  |  |  | 0.757 | | 0.145 - 3.948 | 0.742 |
| **PHT requiring NO therapy** | Birthweight (pc) | 0.807 | 0.599 - 1.089 | 0.161 | 1.500 | | 0.285 - 7.902 | 0.633 |
|  | *GA CPR < 1* (weeks) | *0.713* | *0.521 - 0.975* | ***0.034*** | 0.995 | | 0.873 - 1.135 | 0.941 |
|  | Maternal age (years) | 0.883 | 0.754 - 1.033 | 0.119 | 0.741 | | 0.536 - 1.026 | 0.071* |
|  | Maternal BMI (kg/m^2^) | 1.004 | 0.882 - 1.143 | 0.954 | 0.885 | | 0.730 - 1.025 | 0.094 |
|  | gender: female | 0.647 | 0.115 - 3.640 | 0.621 | 0.995 | | 0.873 - 1.135 | 0.941 |
|  | PETN |  |  |  | 0.619 | | 0.104 - 3.670 | 0.597 |
| **Gastrointestinal complications requiring surgery** | GA at birth (days) | 0.972 | 0.921 - 1.025 | 0.296 | 1.991 | | 0.334 - 11.871 | 0.450 |
|  | *Birthweight (pc)* | *0.701* | *0.499 - 0.986* | ***0.041*** | 0.984 | | 0.937 - 1.034 | 0.534 |
|  | *GA CPR < 1* (weeks) | *0.601* | *0.388 - 0.931* | ***0.023*** | 0.752 | | 0.552 - 1.024 | 0.070* |
|  | Maternal age (years) | 1.148 | 0.977 - 1.349 | 0.094 | 0.670 | | 0.457 - 0.980 | **0.039** |
|  | Maternal BMI (kg/m^2^) | 1.061 | 0.916 - 1.229 | 0.427 | 3.061 | | 0.378 - 24.780 | 0.431 |
|  | gender: female | 6.379 | 0.639 - 63.687 | 0.114 | 0.724 | | 0.004 - 117.935 | 0.736 |
|  | PETN |  |  |  | 1.117 | | 0.971 - 1.286 | 0.122 |
| **BPD** | *GA at birth* (days) | *0.937* | *0.889 - 0.989* | ***0.017*** | 1.045 | | 0.913 - 1.196 | 0.523 |
|  | GA CPR < 1 (weeks) | 0.838 | 0.602 - 1.165 | 0.292 | 2.973 | | 0.403 - 21.942 | 0.285 |
|  | Maternal age (years) | 0.998 | 0.870 - 1.145 | 0.980 | 0.561 | | 0.093 - 3.377 | 0.528 |
|  | Maternal BMI (kg/m^2^) | 1.003 | 0.879 - 1.145 | 0.964 | 0.939 | | 0.890 - 0.991 | **0.022** |
|  | gender: female | 0.725 | 0.144 - 3.649 | 0.696 | 0.813 | | 0.571 - 1.159 | 0.253 |
|  | PETN |  |  |  | 1.006 | | 0.875 - 1.157 | 0.933 |
| **ROP** | *GA at birth* (days) | *0.859* | *0.773 - 0.954* | ***0.005*** | 1.005 | | 0.879 - 1.148 | 0.945 |
|  | APGAR score at 5 minutes | 1.082 | 0.347 - 3.373 | 0.892 | 0.763 | | 0.150 - 3.888 | 0.745 |
|  | Maternal age (years) | 0.943 | 0.790 - 1.126 | 0.519 | 0.670 | | 0.139 - 3.236 | 0.618 |
|  | Maternal BMI (kg/m^2^) | 1.139 | 0.906 - 1.433 | 0.265 | 0.858 | | 0.722 - 0.954 | **0.005** |
|  | gender: female | 0.217 | 0.016 - 2.899 | 0.248 | 1.087 | | 0.346 - 3.411 | 0.886 |
|  | PETN |  |  |  | 0.936 | | 0.768 - 1.142 | 0.516 |
| **Discharge supported by home medical equipment** | *GA at birth* (days) | *0.945* | *0.905 - 0.987* | ***0.010*** | 1.136 | | 0.901 - 1.433 | 0.280 |
|  | Maternal age (years) | 1.043 | 0.930 - 1.170 | 0.475 | 0.229 | | 0.016 - 3.273 | 0.277 |
|  | Maternal BMI (kg/m^2^) | 1.019 | 0.900 - 1.155 | 0.763 | 1.237 | | 0.108 - 14.117 | 0.864 |
|  | gender: female | 0.581 | 0.103 - 3.277 | 0.539 | - 0.486 | | (- 1.153) - 0.180 | 0.139* |
|  | PETN |  |  |  | - 4.090 | | (- 6.914) - (- 1.267) | **0.008** |
| **Length of stay on NICU - discharge (Survivors)** | *GA at birth* (days) | *- 0.792* | *- 1.547 - - 0.037* | ***0.041*** | - 6.294 | | (- 16.468) - 3.881 | 0.204 |
|  | *GA CPR < 1* (weeks) | *- 3.630* | *- 7.024 - - 0.235* | ***0.038*** | 21.121 | | (- 10.197) - 52.439 | 0.169 |
|  | APGAR score at 5 minutes | - 3.134 | - 15.107 - 8.839 | 0.583 | *44.542* | | 8.568 - 80.516 | **0.019*** |
|  | Mode of delivery (secondary vs. primary C-section) | - 2.174 | - 33.278 - 28.929 | 0.883 | *2.283* | | (- 17.222) - 21.788 | 0.804 |
|  | *DV: reverse flow* | *37.174* | *- 5.877 - 80.226* | *0.085* | *0.540* | | (- 0.850) - 1.931 | 0.416 |
|  | Stressed obstetric history (yes) | *12.010* | - 9.814 - 33.834 | 0.258 | - 0.749 | | (- 2.191) - 0.693 | 0.282 |
|  | Maternal age (years) | - 0.232 | - 1.754 - 1.291 | 0.749 | 5.430 | | (- 10.521) - 21.382 | 0.475 |
|  | Maternal BMI (kg/m^2^) | 0.093 | - 1.468 - 1.654 | 0.093 | - 21.141 | | (- 37.429) - (- 4.853) | **0.015*** |
|  | gender: female | 5.659 | - 13.672 - 24.990 | 0.540 | 0.18 | | 0.865 - 0.973 | **0.004*** |
|  | PETN |  |  |  | 0.570 | | 0.406 - 0.801 | **0.00*1** |
